# Constraints of Metabolic Energy on the Number of Synaptic Connections of Neurons and the Density of Neuronal Networks

**DOI:** 10.3389/fncom.2018.00091

**Published:** 2018-11-20

**Authors:** Ye Yuan, Hong Huo, Peng Zhao, Jian Liu, Jiaxing Liu, Fu Xing, Tao Fang

**Affiliations:** ^1^Department of Automation, Shanghai Jiao Tong University, Shanghai, China; ^2^Key Laboratory of System Control and Information Processing, Ministry of Education, Shanghai, China

**Keywords:** neuronal networks, network topology, synaptic organization rules, metabolic energy, energy balance, computational model

## Abstract

Neuronal networks in the brain are the structural basis of human cognitive function, and the plasticity of neuronal networks is thought to be the principal neural mechanism underlying learning and memory. Dominated by the Hebbian theory, researchers have devoted extensive effort to studying the changes in synaptic connections between neurons. However, understanding the network topology of all synaptic connections has been neglected over the past decades. Furthermore, increasing studies indicate that synaptic activities are tightly coupled with metabolic energy, and metabolic energy is a unifying principle governing neuronal activities. Therefore, the network topology of all synaptic connections may also be governed by metabolic energy. Here, by implementing a computational model, we investigate the general synaptic organization rules for neurons and neuronal networks from the perspective of energy metabolism. We find that to maintain the energy balance of individual neurons in the proposed model, the number of synaptic connections is inversely proportional to the average of the synaptic weights. This strategy may be adopted by neurons to ensure that the ability of neurons to transmit signals matches their own energy metabolism. In addition, we find that the density of neuronal networks is also an important factor in the energy balance of neuronal networks. An abnormal increase or decrease in the network density could lead to failure of energy metabolism in the neuronal network. These rules may change our view of neuronal networks in the brain and have guiding significance for the design of neuronal network models.

## Introduction

The cognitive functions of the brain are performed through interactions among thousands of neurons, and neuronal networks in the brain are the structural basis of such interactions (Gu et al., 2015; Medaglia et al., 2015). Understanding the general synaptic organization rules by which neurons are organized into neuronal networks is essential for gaining further insight into brain cognitive functions. However, most previous works have focused on changes in synaptic connections between neurons but seldom on the network topology of these connections. Over the past decades, extensive studies have shown that neurons in neuronal networks interact with each other through synaptic connections, and brain cognitive functions such as the formation of memories and the learning of actions, are closely related to the changes in synaptic connections (Neves et al., 2008; Sweatt, 2016). Based on vast experimental observations, researchers have proposed various theories to interpret these changes. The most influential theory is Hebb's postulate, which studies changes in synaptic connections as a function of pre- and post-synaptic neuronal activities (Hebb, 1949; Caporale and Dan, 2008). Nevertheless, increasing studies indicate that in addition to pre- and post-synaptic neuronal activities, other factors can also modulate the changes in synaptic connections, such as glia and neuromodulators (Picciotto et al., 2012; Corty and Freeman, 2013; Mitsushima et al., 2013; De Pittà et al., 2016). These findings raise the question of whether there exist any general synaptic organization rules that potentially guide the changes in synaptic connections. Growing evidence suggests that metabolic energy may be a unifying principle governing neuronal activities (Laughlin, 2001; Niven and Laughlin, 2008; Hasenstaub et al., 2010; Yu and Yu, 2017), which naturally leads to the inference that changes in synaptic connections are also under the governance of metabolic energy. Synaptic transmission and dendritic integration are two main metabolically expensive steps in neuronal information processing, and any changes in synaptic connections can result in fluctuations of their energy consumption (Attwell and Laughlin, 2001; Howarth et al., 2012; Yu et al., 2017). In particular, synaptic transmission may play very important roles in regulating collective neuronal activities, such as synchronization (Wang et al., 2011; Guo et al., 2012), firing patterns and dynamics (Guo et al., 2016b), and resonance (McDonnell and Abbott, 2009). To ensure normal information processing in neurons, the energy balance between synaptic transmission and dendritic integration should be maintained regardless of how synaptic connections change. Obviously, due to the close coupling between synaptic activities and metabolic energy, the network topology of synaptic connections should follow some general synaptic organization rules so that constructed neuronal networks match their energy metabolism.

In this paper, from the perspective of energy metabolism, a computational model is developed to investigate the general synaptic organization rules for neurons and neuronal networks. In this model, all regulatory actions on synaptic connections are performed under energy constraints proposed to maintain the energy balance between synaptic transmission and dendritic integration in all neurons of the neuronal networks. We first study the effects of metabolic energy on synaptic connections in individual neurons and neuronal networks and then discuss the general synaptic organization rules for neurons and neuronal networks under the effects of metabolic energy based on the simulation results. Some studies argue that in addition to synaptic connections, neurons can transmit information through field coupling (Xu et al., 2018), which also consumes energy. And that, body temperature is closely related to energy metabolism in the brain (Yu et al., 2014). Therefore, based on the conclusions in this paper, implicit influences of field coupling and body temperature on the energy metabolism will be discussed in the end.

## Methods

### Model of biological neuronal networks in the brain

**Figure 1:**
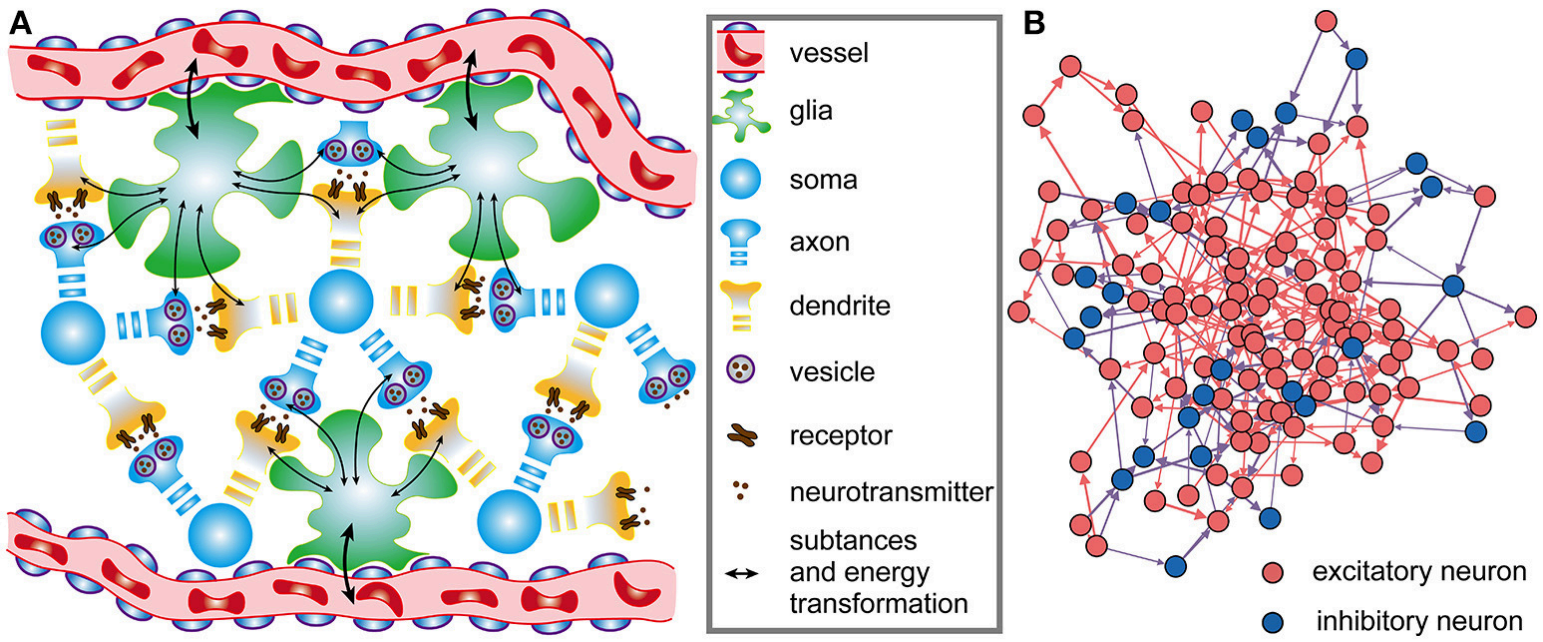
Architectures of biological neuronal networks in the brain**. (A)** Biological networks in the brain contain many neurons, glia, and adjacent capillaries. These neurons are not only densely connected to each other in a complex recurrent way but are also tightly coupled to glia. Glia can continuously transport oxygen and energy substrates within capillaries to neurons and then remove metabolic wastes from neurons. **(B)** From the perspective of graph theory, neurons as well as the synaptic connections between them can be summarized and represented as a set of nodes and edges forming a network. In the network, red, and blue circles represent excitatory and inhibitory neurons, respectively. Synaptic connections between excitatory neurons are indicated by red arrows, and synaptic connections between excitatory and inhibitory neurons are indicated by blue arrows. Note that synaptic connections between inhibitory neurons do not exist in the network. All neurons in the network receive stimuli from outside the network. For simplicity, only a portion of the neurons and synaptic connections are drawn in the figure.

The architecture of neuronal networks undoubtedly plays a strong role in determining neuronal activity and energy metabolism, and vice versa (Raichle and Mintun, 2006; Smith et al., 2009). In neuronal networks, neurons are closely coupled with glia, and adjacent capillaries provide them sufficient metabolic substrates and remove metabolic wastes (Magistretti and Allaman, 2015) (Figure 1A). Unlike artificial neural networks in a simple feed-forward structure, brains are characterized by highly recurrent neuronal networks that can exhibit abundant activity patterns and perform complex computations (Sussillo and Abbott, 2012; Nessler et al., 2013; Sussillo, 2014). Therefore, a small-scale and recurrent neuronal network is built here to simulate the spiking activities in biological neuronal networks under the effects of metabolic energy.

Consider a dynamic network of *N* neurons described by the following differential equation:

(1) $${\dot{x}}_{j}=\psi (x_{j})+\sum_{i=1}^{N}w_{ij}\cdot \varepsilon (x_{i})+\sum_{k=1}^{M}b_{kj}\cdot u_{k},j=1,2,...,N$$

where *x*_*j*_ captures the state of the *j-th* neuron, and ψ(·) is a non-linear function that describes the dynamics of the *j-th* neuron itself; *w*_*ij*_ denotes the weight of a directed connection from the *i-th* neuron to the *j-th* neuron, and its range is 0 ≤ *w*_*ij*_ ≤ 1; ε(·) defines the output spiking signal of the *i-th* neuron; *M* is the number of external signal sources of the network; *b*_*kj*_ is the weight of a directed connection from the *k-th* external signal source to the *j-th* neuron, and *u*_*k*_ denotes the spiking signal produced by the *k-th* external signal source. Obviously, Equation (1) defines the wiring diagram of the network. In the network, the neurons simultaneously receive signals from other neurons within the network as well as signals from sources outside the network, and the states of the neurons can change over time. For the individual neurons in the network, the leaky integrate-and-fire (I&F) neuron model is used here to simulate their activities (see Supplementary Materials) (Izhikevich, 2004). It is assumed that the neuronal network contains *N*^*E*^ excitatory neurons and *N*^*I*^ inhibitory neurons, i.e., *N* = *N*^*E*^ + *N*^*I*^ (Figure 1B). In the network, excitatory neurons can be connected with any neurons, but inhibitory neurons can only be connected with excitatory neurons. That is, there are three types of synaptic connections in the network: those from excitatory neurons to excitatory neurons, those from excitatory neurons to inhibitory neurons, and those from inhibitory neurons to excitatory neurons. The connections between neurons are unidirectional. For example, if there is a connection from the *i-th* neuron to the *j-th* neuron, the connection from the *j-th* neuron to the *i-th* neuron definitely does not exist. At the beginning of the simulation, whether there are synaptic connections between neurons is random, and the signaling directions and synaptic weights between neurons are also randomly assigned. During the simulation, the directions remain unchanged, but the synaptic weights vary according to the rules proposed later in this paper. According to experimental results, the ratio of the number of excitatory neurons to the number of inhibitory neurons is set to 4:1 in the neuronal network (Markram et al., 2004), and inhibitory neurons are used to balance the activity intensity of the entire network (Hattori et al., 2017). For simplicity, the synaptic weights between excitatory neurons and inhibitory neurons are assumed to remain fixed. In this case, the shaping of the network architectures by metabolic energy is reflected by the changes in synaptic weights between excitatory neurons.

### Energy constraints of neurons in the neuronal network

Because both the number of capillaries throughout the network and the ability of each capillary to transport oxygen and glucose are limited, maintaining the energy balance of various neuronal activities is essential for the normal operation of neurons and neuronal networks (Waterson and Horvath, 2015). Experimental and theoretical studies have shown that the primary processes that consume metabolic energy in neuronal information processing are synaptic transmission and dendritic integration (Alle et al., 2009; Howarth et al., 2012; Yuan et al., 2018). For synaptic transmission, there is energy consumption in the presynaptic release, postsynaptic action, and recycling of neurotransmitters, while for dendritic integration, energy consumption occurs during the generation of action potentials. Synaptic transmission and dendritic integration account for ~59 and ~21% of the total signaling-related energy consumption, respectively (Howarth et al., 2012). Maintaining the energy balance between synaptic transmission and dendritic integration is necessary for neurons and their networks to perform normal information processing. To achieve an energy balance similar to real biological neurons, the following energy constraints are imposed on each neuron, as follows:

(2) $$\begin{array}{l} \text{min}\left|\alpha -c\right|,\\ s.t.\alpha =\frac{\int_{t}^{t+\Delta T}E_{j}^{trans}d\tau }{\int_{t}^{t+\Delta T}E_{j}^{trans}d\tau +\int_{t}^{t+\Delta T}E_{j}^{integ}d\tau }\\ E_{j}^{trans}>0,E_{j}^{integ}>0. \end{array}$$

where $E_{j}^{trans}$ and $E_{j}^{integ}$ are the metabolic energies consumed in synaptic transmission and dendritic integration of the *j-th* neuron at time *t*, respectively; α represents the ratio of the metabolic energy consumed in synaptic transmission to the total metabolic energy consumed in synaptic transmission and dendritic integration within Δ*T*. The value of the ratio α is equal to or infinitely close to constant *c* when the metabolic energy consumed in synaptic transmission and dendritic integration are both at normal levels. According to the experimental findings, the constant *c* is fixed at 0.75 (Howarth et al., 2012). Equation (2) implies that the ratio of the metabolic energy consumed in synaptic transmission to the total metabolic energy consumed in synaptic transmission and dendritic integration should approach a constant over any given period of time Δ*T*.

**Figure 2:**
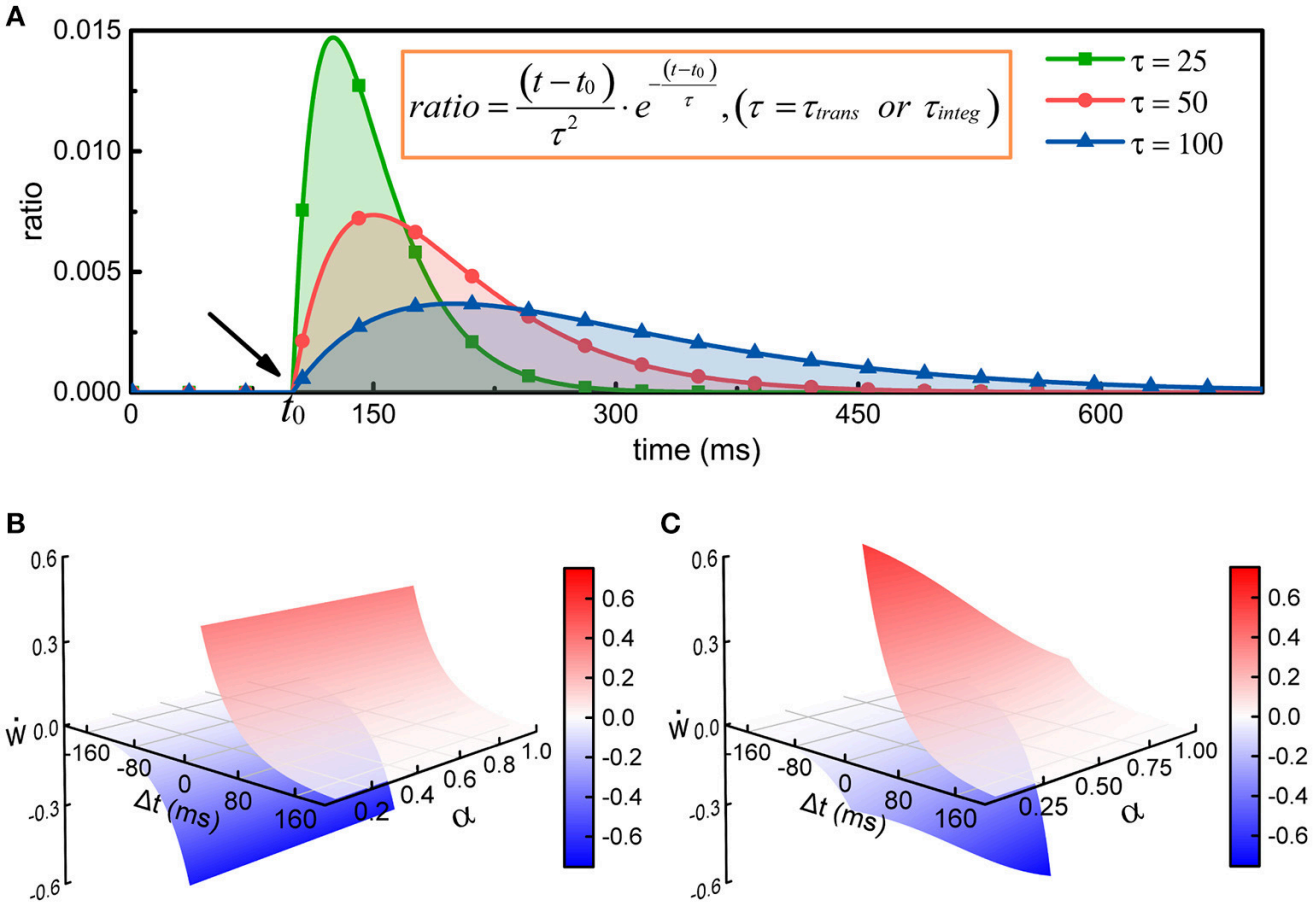
The regulation of synaptic weights by metabolic energy. **(A)** Assuming that a single action potential is transmitted to postsynaptic neurons via synapses or is generated at the axonal initial segment at time *t*_0_, the energy consumption can be described by the curves in the figure. The larger the time constant is, the longer the duration of the process. In addition, no matter what the value of the time constant is, the area enclosed by the curves and horizontal axis can be mathematically proven to equal 1. **(B)** The classical Hebbian postulate modifies synaptic weights based solely on the activities of presynaptic and postsynaptic neurons (see Supplementary Materials). If postsynaptic neurons fire earlier than presynaptic neurons (Δ*t* < 0), the strength of synaptic connections is weakened; otherwise, the strengths are enhanced (Δ*t* > 0). Furthermore, the strengths are not related with the ratio α of the energy consumed in synaptic transmission to the total energy consumed in synaptic transmission and dendritic integration. That is, in the case that Δ*t* is constant, the change in synaptic weights cannot vary with α. **(C)** The strength of synaptic connections is affected by the activities of pre- and post-synaptic neurons and by the metabolic energy of synaptic transmission and dendritic integration. Namely, the changes in synaptic weights not only obey Hebbian rules but also depend on the ratio α. When Δ*t* is constant, the change in synaptic weights is inversely proportional to the ratio α.

In the synaptic transmission phase, assuming presynaptic terminals only contain one release site, each action potential can induce an average of 0.25 synaptic vesicles to release neurotransmitters in presynaptic neurons at 37°C, and the energy expended per vesicle of neurotransmitters released is 1.64 × 10^5^ ATP molecules (Attwell and Laughlin, 2001; Yu et al., 2017). In other words, the metabolic energy consumed in synaptic transmission is $E_{single}^{trans}=4.1\times 10^{4}$ ATP molecules, which corresponds to one action potential arriving at the presynaptic terminal. Thus, the metabolic energy consumed in synaptic transmission can be calculated according to the number of action potentials arriving at presynaptic terminals. Because many biochemical reactions in synaptic transmission, such as synaptic vesicle cycling and the binding of neuromodulators to receptors, are not instantaneously completed, the metabolic energy does not suddenly decrease due to the transmission of action potentials Südhof, 2013; Wu et al., 2014. We multiply the metabolic energy $E_{single}^{trans}$ expended per action potential in synaptic transmission and an exponential function $\varphi_{1}\left(t\right)=\left(t-t_{k}\right)/\tau_{trans}^{2}\cdot e^{-\left(t-t_{k}\right)/\tau_{trans}}$ to reflect the time-varying characteristics of the metabolic energy consumed in synaptic transmission. In addition, the weight of synaptic connections is also an important factor in determining the energy consumption. A larger connection weight means that more information is transmitted and that the energy consumption is greater. Therefore, the energy consumed in the synaptic transmission of the *j-th* neuron at time *t* can be described as follows:

(3) $$E_{j}^{trans}=\sum_{k=1}^{N_{j}^{trans}}\left(E_{single}^{trans}\cdot w_{ij}\cdot \frac{\left(t-t_{k}\right)}{\tau_{trans}^{2}}\cdot e^{-\frac{\left(t-t_{k}\right)}{\tau_{trans}}}\right),\left(t\geq t_{k}\geq 0\right)$$

where $N_{j}^{trans}$ is the number of action potentials propagating to the *j-th* neuron before time *t*, τ_*trans*_ is the time constant of the change in the energy consumption of synaptic transmission, *w*_*ij*_ is the synaptic weight from the *i-th* neuron to the *j-th* neuron, and *t*_*k*_ is the time at which the *k-th* action potential reaches the *j-th* neuron. The greater the time constant τ_*trans*_ is, the longer the duration of the energy consumption process, and conversely, the shorter the duration (Figure 2A). In particular, the integral of φ_1_(*t*) from *t*_*k*_ to positive infinity is always equal to 1, which guarantees that in the case of constant synaptic weights, the total metabolic energy consumed to propagate each action potential remains constant. When *w*_*ij*_ = 1, the total metabolic energy consumed to propagate each action potential is equal to 4.1 × 10^4^ ATP molecules.

In the dendritic integration phase, the ion pumps on membranes must consume a large amount of metabolic energy to recover the concentration balance of ions inside and outside the membranes (Magee, 2000; Spruston, 2008; Stuart and Spruston, 2015). The metabolic energy needed for a single action potential is estimated to be $E_{single}^{integ}=1.2\times 10^{8}$ ATP molecules (Attwell and Laughlin, 2001; Howarth et al., 2012; Yu et al., 2017). Thus, the metabolic energy consumed in dendritic integration can be calculated according to the number of generated action potentials. Similarly, because the active transport of ions is not instantaneous, the corresponding metabolic energy is not immediately consumed. To reflect the time-varying characteristic of metabolic energy consumption, we multiply the metabolic energy $E_{single}^{integ}$ expended per action potential in dendritic integration and an exponential function$\varphi_{2}\left(t\right)=\left(t-t_{l}\right)/\tau_{integ}^{2}\cdot e^{-\left(t-t_{l}\right)/\tau_{integ}}$. Therefore, the energy consumed in the dendritic integration of the *j-th* neuron at time *t* can be described as follows:

(4) $$E_{j}^{integ}=\sum_{l=1}^{N_{j}^{integ}}\left(E_{single}^{integ}\cdot \frac{\left(t-t_{l}\right)}{\tau_{integ}^{2}}\cdot e^{-\frac{\left(t-t_{l}\right)}{\tau_{integ}}}\right),\left(t\geq t_{l}\geq 0\right)$$

where $N_{j}^{integ}$ is the number of action potentials generated by the *j-th* neuron before time *t*, τ_*integ*_ is the time constant of the change in the energy consumption of dendritic integration, and *t*_*l*_ is the time at which the *l-th* action potential is generated by the *j-th* neuron. The greater the time constant τ_*integ*_ is, the longer the duration of the energy consumption process, and conversely, the shorter the duration (Figure 2A). Similarly, the integral of φ_2_(*t*) from *t*_*l*_ to positive infinity is always equal to 1, which guarantees that the total energy consumed to generate each action potential is equal to 1.2 × 10^8^ ATP molecules.

Equations (3, 4) have similar characteristics, and they both contain an exponential function. In fact, the curves determined by the exponential functions show a tendency to rise first and then decrease, and the time constant determines the rise and decay times of the curves (Figure 2A). The exponential functions are usually used to describe the changes in postsynaptic membrane potentials and ion currents induced by spikes in neurons (Gerstner, 1995; Bohte et al., 2002). Here, the exponential functions are used to describe the time-varying characteristics of the energy consumption when calculating the metabolic energies consumed in synaptic transmission and dendritic integration. Because the physical units of the time constants (τ_*trans*_ and τ_*integ*_) and time variables (*t*_*k*_, *t*_*l*_, and *t*) are both second, the calculated results of the exponential functions are dimensionless values. The weight *w*_*ij*_ is also a dimensionless value. Therefore, the calculated results of Equations (3, 4) have the same physical units as the energy constants ($E_{j}^{trans}$ and $E_{j}^{integ}$). For convenience, the default values of some parameters in the network model are summarized in Table 1.

**Table 1:** The default values of several parameters.

**Symbol**	**Meaning**	**Value**
$E_{single}^{trans}$	The energy expended per action potential in synaptic transmission	4.1 × 10^4^ ATP molecules
$E_{single}^{integ}$	The energy expended per action potential in dendritic integration	1.2 × 10^8^ ATP molecules
*c*	When the neurons are in energy balance, the ratio of the energy consumed in synaptic transmission to the total energy consumed in synaptic transmission and dendritic integration within Δ*T*	0.75
Δ*T*	Sliding time window for the calculation of the energy consumption	5 s
τ_*trans*_	The time constant of the change in the energy consumption for synaptic transmission	20 ms
τ_*integ*_	The time constant of the change in the energy consumption for dendritic integration	100 ms
λ	A dimensionless constant that specifies the degree of influence of metabolic energy on neuronal activities	300
*f*	The frequency of external spiking signals	10 Hz

### Synaptic learning rules with the energy constraints

Synaptic transmission and dendritic integration, two metabolically expensive signaling-related activities in neurons, require a large amount of metabolic energy to fuel, which also means that they are strongly regulated by limited metabolic energy (Attwell and Laughlin, 2001; Howarth and Peppiatt, 2010; Howarth et al., 2012). Although the specific mechanisms for achieving the regulation of synaptic transmission and dendritic integration by metabolic energy have not been completely revealed, their energy consumption is undoubtedly closely related to synaptic connections, specifically, the number of synaptic connections and the strength of synaptic connections. For example, if the number of synaptic connections to a single neuron is reduced and the strength of the synaptic connections is weakened, the activity intensity of postsynaptic neurons will decrease, and the metabolic energy consumed in synaptic transmission and dendritic integration will be reduced accordingly. Therefore, to reflect the close relationship between metabolic energy and synaptic connections, we refer to the three-factor synaptic plasticity rules (Frémaux and Gerstner, 2016; Foncelle et al., 2018) and then combine the synaptic learning rules (STDP) with the energy constraints, as follows:

(5) $$\dot{w}=f\left(\alpha \right)\cdot H\left(pre,\;post\right)$$

(6) $$f\left(\alpha \right)=\frac{2}{1+e^{\lambda }{}^{\cdot \frac{\Delta t}{\left|\Delta t\right|}\cdot \left(\alpha -c\right)}},\left(\lambda >0\right)$$

where ẇ describes the rate of change of synaptic weights, and *pre* and *post* denote the spike trains of pre- and post-synaptic neurons, respectively. The variable α and constant *c* are the same as in Equation (2). The function *H*(·) describes how pre- and postsynaptic spike trains determine the changes in synaptic weights (see Supplementary Materials), and *f*(·) is a function that describes the regulation of the changes in synaptic weights by metabolic energy. λ is a dimensionless constant that specifies the degree of influence of metabolic energy on neuronal activities, and Δ*t* is equal to the latest spike time of postsynaptic neurons minus that of presynaptic neurons. It can be found that when neurons reach an energy balance, our synaptic learning rules can be changed into the classical STDP learning rules. In this situation, the increment or decrement in synaptic weights depends only on the spiking activities of pre- and post-synaptic neurons (Figure 2B). In fact, the largest difference between two synaptic learning rules is the way in which the value of the function *f*(·) is determined. In our synaptic learning rules, this value is given based on the energy consumption ratio between synaptic transmission and dendritic integration. According to our rules, when the ratio α deviates from the constant *c*, the changes in synaptic weights will be regulated by metabolic energy, and the greater the deviation is, the stronger the regulation. More specifically, when the ratio α is larger than the constant *c*, the increment and decrement in the synaptic weights derived from the STDP rules will be reduced and amplified, respectively. Conversely, in the case that α is smaller than *c*, the increment and decrement in the synaptic weights derived from the STDP rules will be amplified and reduced, respectively (Figure 2C). Based on these analyses, it is clear that the changes in synaptic weights are inversely proportional to the ratio α, which is similar to negative feedback in engineering (Davis, 2006).

Extensive studies have shown that the negative feedback widely occurs in organisms and helps organisms maintain homeostasis (Davis, 2006; Turrigiano, 2007). Energy balance is an essential part of neuronal homeostasis. Thus, although the specific biological mechanisms through which metabolic energy exerts feedback control on neuronal activities have not been fully revealed, there is no doubt that all the changes in synaptic connections must maintain the energy balance in neurons. Moreover, increasing studies also show that metabolic energy is a unifying principle governing various neuronal activities (Laughlin, 2001; Bialek et al., 2006; Niven and Laughlin, 2008; Hasenstaub et al., 2010). From this perspective, the combination of the energy constraints and synaptic learning rules in our model is biologically reasonable. The proposed model is implemented in MATLAB, in which GPU is adopted for parallel operations; the code is available upon request.

## Results

### Effects of metabolic energy on the synaptic connections in individual neurons

**Figure 3:**
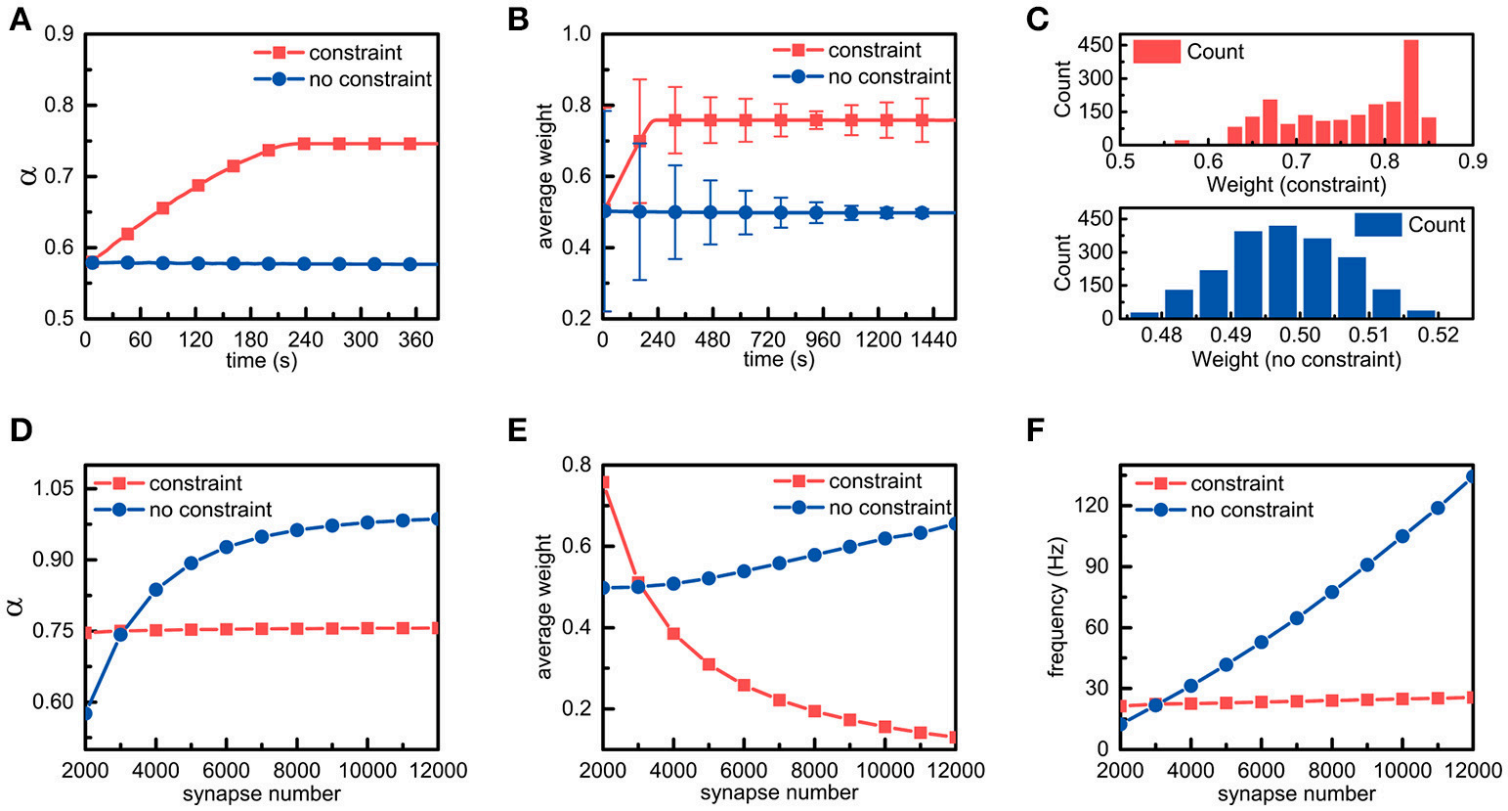
Simulation results of a single neuron with energy constraints**. (A)** In the presence and absence of energy constraints, the ratios of the energy consumed in synaptic transmission to the total energy consumed in synaptic transmission and dendritic integration are recorded and plotted in the corresponding curves, respectively. It is noted that during the simulation, the frequency of the external stimulus is set to 10 Hz unless otherwise specified. **(B)** In the simulation process, the synaptic weights in both cases are sampled every 7.68 s. In addition, their averages and standard deviations are calculated, respectively. **(C)** After the synaptic weights in both cases become stable, the corresponding synaptic weight distribution is drawn. Chi-square results show that the stable synaptic weights without the energy constraints roughly agree with a normal distribution. **(D–F)** In the case of different numbers of synaptic connections, the ratio α, the average of synaptic weights and the spike frequency in the presence and absence of the energy constraints are recorded. It should be noted that these data are recorded when the ratio α becomes stable.

We assume that an individual neuron has *m* synaptic connections, and their weights are represented by *w*_*i*_(0 < *i* ≤ *m*). As external stimuli, *m* action potential sequences with a frequency of *f* Hz are transmitted to the neuron via synaptic connections with different weights, respectively. For simplicity, the number of synaptic connections in a single neuron is set to 2,000. In addition to the energy constraints, all parameters are fixed in the simulations. It can be found from Figure 3A that in the presence of the energy constraints, the ratio of the energy consumed in synaptic transmission to the total energy consumed in synaptic transmission and dendritic integration gradually increases and then stabilizes around the set point, while the ratio in the absence of the energy constraints almost remains unchanged. In addition, the ratio in the presence of the energy constraints is obviously larger than that in the absence of the energy constraints, which is consistent with experimental findings that neurons consume more metabolic energy in synaptic transmission (~59%) than in dendritic integration (~21%).

There are only three ways in which the energy consumption of synaptic transmission and dendritic integration can be altered, namely, altering the number of synaptic connections, the synaptic weights, and the frequency of external stimulus. Given that the number of synaptic connections and the frequency of the external stimulus are fixed in the simulation, only altering synaptic weights can lead to changes in energy consumption. Thus, we sample the synaptic weights obtained in the simulations in both cases and statistically analyse these data. As expected, the differences in the synaptic weights are surprisingly obvious in both cases. From Figure 3B, it can be found that the changes in the trends of the average weights of all synaptic connections in neurons in both cases are the same as their respective ratio α, and the average synaptic weight in the presence of the energy constraints is obviously larger than that in the absence of the energy constraints. After the average synaptic weights in both cases become stable, the average weights of the former and the latter are approximately equal to 0.758 and 0.498, respectively. Furthermore, although the average synaptic weights in both cases become stable, their standard deviations still change over time. Clearly, the standard deviation in the presence of the energy constraints first decreases and then slightly increases, while the standard deviation in the absence of the energy constraints always decreases (Figure 3B). To further investigate the distribution of the synaptic weights, after the synaptic weights in both cases become stable, the corresponding synaptic weight distribution is drawn in Figure 3C. It can be found that there exists a large difference in the synaptic weight distributions in both cases. The synaptic weight distribution in the presence of the energy constraints has no obvious regularity and the distribution range is ~0.55~0.85, while the synaptic weight distribution in the absence of the energy constraints roughly follows a normal distribution. The largest difference is that the synaptic weights of neurons with the energy constraints are overall larger than the synaptic weights in the absence of the energy constraints. These simulation results suggest that the energy constraints in our model can achieve an energy balance between synaptic transmission and dendritic integration by increasing or decreasing the synaptic weights in individual neurons.

To study the effects of metabolic energy on the synaptic connections in individual neurons more comprehensively, we set different numbers of synaptic connections *m* to investigate the changes in the ratio α, the average of synaptic weights and the spike frequency of neurons in the presence and absence of the energy constraints. The ratio α in both cases is recorded after it is roughly stable. As shown in Figure 3D, in the presence of the energy constraints, the ratio α can always be maintained stable near the set point as the number of synaptic connections increases, while the ratio α in the absence of the energy constraints quickly increases as the number of synaptic connections increases. With the energy constraints, neurons can maintain an energy balance even if the number of synaptic connections changes. Without the energy constraints, although a greater number of synaptic connections can simultaneously cause an increase in the energy consumption of synaptic transmission and dendritic integration, the increase in energy consumption of synaptic transmission is faster than that of dendritic integration. The average weights of the synaptic connections as well as the spike frequencies of neurons in both cases are also recorded after they are roughly stable. Their average weights vary non-linearly with the number of synaptic connections, but the change in tendencies are diametrically opposite (Figure 3E). The average synaptic weight in the presence of the energy constraints sharply decreases as the number of synaptic connections increases, whereas the average synaptic weight in the other case increases slowly with the number of synaptic connections. In addition, as the number of synaptic connections increases, the spike frequency of neurons with the energy constraints remains almost unchanged, while the spike frequency in the absence of the energy constraints linearly rises (Figure 3F). From the simulation results, it can be concluded that with the energy constraints, no matter how the number of synaptic connections changes, neurons can always adjust synaptic weights accordingly to keep the energy balance. This suggests that the energy balance in individual neurons is determined by both the number and weights of synaptic connections rather than any one of them. In addition, the spike frequency is generally regarded as an indicator of the activity intensity as well as the energy consumption of neurons. Larger spike frequencies means more intense neuronal activities and more energy consumption. From this perspective, the neurons in the presence of the energy constraints can not only balance the energy consumption between synaptic transmission and dendritic integration but also keep the total energy consumption of neurons unchanged even if the external stimulus intensely fluctuates.

### Effects of metabolic energy on the synaptic connections in the neuronal network

Neurons are crucial components of neuronal networks, and their energy metabolism properties have a great influence on the energy consumption of signaling-related activities in neuronal networks. We show above how metabolic energy strongly affects the synaptic connections and the activity intensities in individual neurons. Here, to investigate the effects of metabolic energy on the synaptic connections in neuronal networks, we set the number of excitatory and inhibitory neurons in the neuronal network to 500 and 125, respectively, and simulate the spiking activities in the entire neuronal network. Action potential sequences with a frequency of *f* Hz are transmitted to all neurons via synaptic connections with different weights. These action potential sequences are regarded as external stimuli that ensure the neurons in the neuronal network remain active during the simulation.

**Figure 4:**
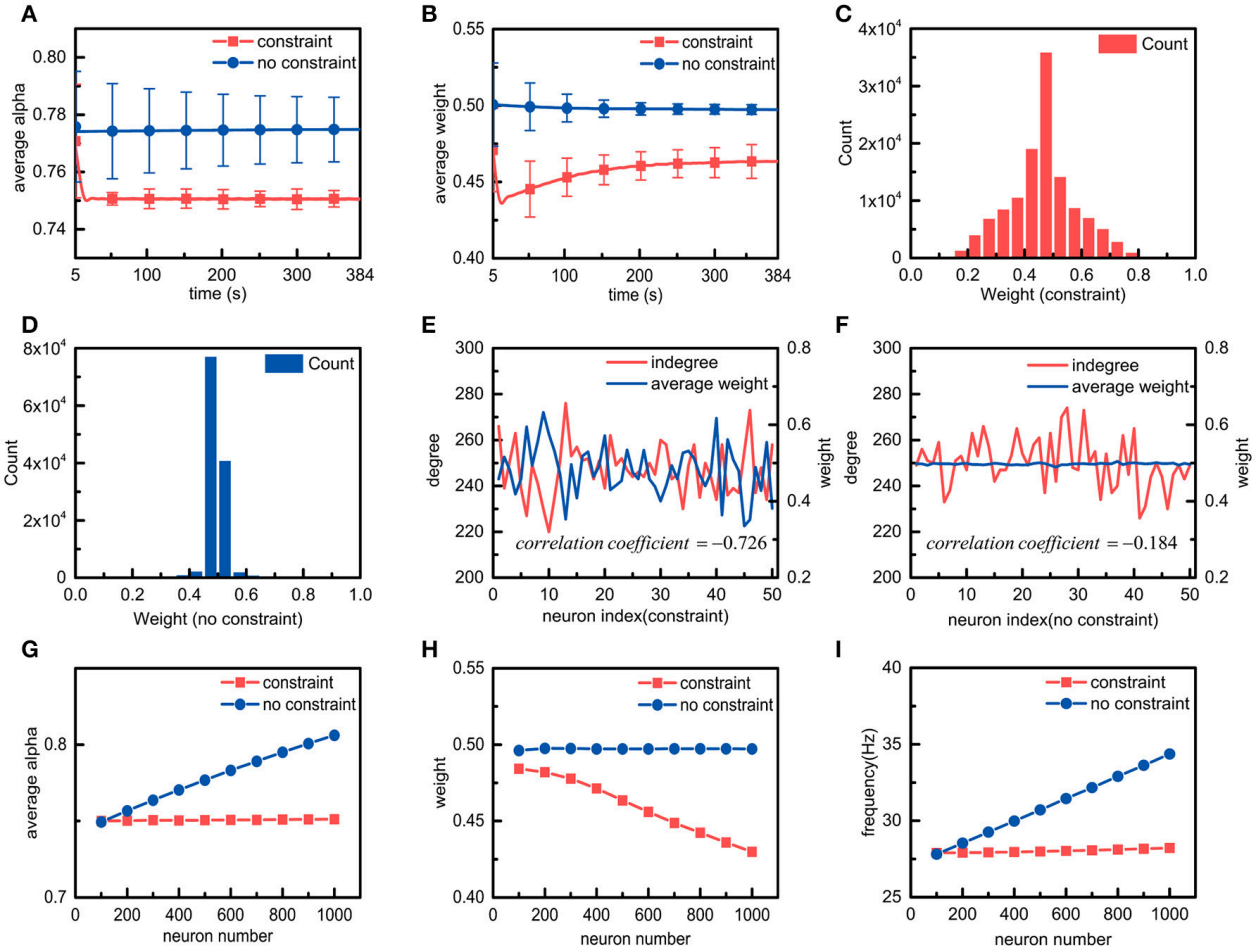
Simulation results of neuronal networks with and without energy constraints. **(A,B)** in the presence or absence of the energy constraints, the average ratio α and the average weight of the neuronal network change over time. **(C,D)** in the presence or absence of the energy constraints, the synaptic weight distribution when the average ratio and the average weight of the neuronal network become stable. **(E,F)** in the presence or absence of the energy constraints, the relations between the number of synaptic connections and the average weights of a portion of neurons when both networks reach stability. In both cases, the correlation coefficients between the number of synaptic connections and the average synaptic weights are also calculated. The closer the absolute value of the correlation coefficient is to 1, the stronger the correlation between the variables. **(G–I)**, the average ratio, the average weight, and the frequency of the neuronal network vary with the number of neurons in the neuronal network.

The effects of metabolic energy on the basic properties of the synaptic connections in the neuronal network, such as the average ratio $\bar{\alpha }$, the average synaptic weight, and the synaptic weight distribution of the neuronal network, are first investigated. The average ratio $\bar{\alpha }$ and the average synaptic weight of the neuronal network can be calculated according to the ratios of all the neurons as well as all the synaptic weights in the neuronal network. When the average ratio approaches the set point, the neuronal network is considered to reach the energy balance between synaptic transmission and dendritic integration. As shown in Figure 4A, during the simulation, the average ratio $\bar{\alpha }$ of the neuronal network with the energy constraints quickly approaches and stabilizes near the set point, whereas the average ratio in the absence of the energy constraints remains almost unchanged and is always stable at a value higher than the set point. In addition, the standard deviation of the average ratio $\bar{\alpha }$ in the presence of the energy constraints is significantly smaller than the standard deviation in the absence of the energy constraints. In fact, even if the frequency of the external stimulus received by each neuron in the network is the same, there are still large differences in the spiking activities of neurons due to the diversities in synaptic weights, spiking times, and synaptic connections in the network. Despite this, the average ratio $\bar{\alpha }$ of the neuronal network with the energy constraints still approaches the set point, i.e., the energy balance between synaptic transmission and dendritic integration is achieved. These results suggest that the energy constraints not only can achieve the energy balance of individual neurons but also that of neuronal networks. The changes in the average synaptic weights of the neuronal network in both cases are shown in Figure 4B. It can be seen that the average synaptic weight of the constrained neuronal network is obviously smaller than that of the unconstrained neuronal network. The former decreases first and then gradually increases, and it eventually stabilizes at approximately 0.46, while the latter is always stable at approximately 0.5. As we know, the average synaptic weight can only reflect the overall strength of the synaptic connections in the neuronal network, but it cannot show the differences among synaptic weights. To study the differences in synaptic weights in the neuronal network in both cases, the synaptic weight distributions after the average synaptic weights in the neuronal networks become stable are shown in Figures 4C,D, respectively. The synaptic weight distribution in the constrained neuronal network is centered around the average weight of 0.46, while the distribution of the synaptic weights in the unconstrained neuronal network is centered around the average weight of 0.5. Moreover, the synaptic weight distribution range in the former network is much wider than that of the latter network. It is worth noting that the initial synaptic weights in the neuronal network conform to a uniform distribution between 0 and 1, and the initial average synaptic weight of the neuronal network should be equal to 0.5. Thus, the simulation results again confirm that the energy constraints can achieve the energy balance in the network by changing the overall synaptic weights. In the simulation for the neuronal network, it can be clearly found that there are significant relations between the standard deviation of the synaptic weights and the energy constraints. To understand why the standard deviation of the synaptic weights in the constrained network is greater than that in the unconstrained network, we randomly select 50 neurons from the neuronal network in both cases and calculate the number of their synaptic connections and their average synaptic weights (Figures 4E,F). Obviously, in the presence of the energy constraints, the number of synaptic connections is inversely related to the average synaptic weights, while in the absence of the energy constraints, there is no obvious relation between the number of synaptic connections and the average synaptic weights. As we know, neurons in the neuronal network have different numbers of synaptic connections. Therefore, when neurons in the network reach the energy balance, the synaptic weights differ greatly, resulting in a larger standard deviation.

After understanding the effects of metabolic energy on the basic properties of the synaptic connections in the neuronal network, we focus on a very important issue, namely, the relationship between the number of neurons and the synaptic connections in the neuronal network in the presence of the energy constraints. We simulate the spiking activities of the neuronal network with different numbers of neurons (Figures 4G–I). In addition to the average ratio and the average synaptic weight, the average spike frequency is also calculated according to the spike frequencies of all the neurons in the network. In the presence of the energy constraints, as the number of neurons in the neuronal network increases, the average ratio and the average spike frequency of the neuronal network remain almost unchanged, but the average synaptic weight gradually decreases. However, in the absence of the energy constraints, the average ratio, the average synaptic weight, and the average spike frequency exhibit the diametrical opposite of those in the presence of the energy constraints. The results suggest that with the energy constraints, all of the neurons in the neuronal network can maintain the energy balance between synaptic transmission and dendritic integration no matter how the number of neurons in the neuronal network changes. In addition, it can also be found that with the energy constraints, the number of neurons in the neuronal network is inversely proportional to the average synaptic weight of the network. In fact, the neuronal network defined in the paper adopts a full connection strategy to organize all neurons. That is, the increase in the number of neurons in the neuronal network means that the number of synaptic connections per neuron increases. We elaborate the relation between the number and the average weight of synaptic connections in the simulation of individual neurons with the energy constraints. Obviously, in the presence of the energy constraints, the relation between the number of neurons in the neuronal network and the average synaptic weight of the network is actually an extension of the relation between the number and the average weight of synaptic connections in neurons. Similar to individual neurons, the average spike frequency of neuronal networks can also be used to characterize the energy consumption of neuronal networks. Figure 4I shows that in the presence of the energy constraints, the average spike frequency of the neuronal network remains stable as the number of neurons in the network increases, which means that its energy consumption remains almost constant. For the neuronal network without the energy constraints, as the number of neurons in the network increases, the average spike frequency cannot stabilize, and the energy consumption is not constant.

## Discussion and conclusion

### Counterbalances between the number and strength of synaptic connections in neurons

The study described in this paper aims to explore the general synaptic organization rules for neurons and neuronal networks. With our proposed computational model, the basic properties of the synaptic connections of individual neurons are first explored, such as the average of synaptic weights, the number of synaptic connections, and the ratio of the energy consumed in synaptic transmission to the energy consumed in synaptic transmission and dendritic integration. We find that while keeping the ratio of neurons constant, the number of synaptic connections is inversely proportional to the average of the synaptic weights. As defined, the ratio is actually a measure of the energy balance between synaptic transmission and dendritic integration in neurons. Maintaining the metabolic energy balance in neurons is a prerequisite for the normal function of neurons, which means that the ratio is roughly constant in neurons under normal physiological conditions. Therefore, the number of synaptic connections may be inversely related to the strength of synaptic connections in real biological neurons, and maintaining an inverse correlation between the number and strength of synaptic connections may be a strategy by which neurons maintain their own energy balance.

The quantitative relationship among the number of synaptic connections, the average of synaptic weights, and the ratio in the simulation results can be mathematically described as follows:

(7) $$m\cdot \bar{w}\propto \frac{\Delta E^{trans}}{\Delta E^{trans}+\Delta E^{integ}}=\alpha$$

where *m* represents the number of synaptic connections, $\bar{w}$ represents the average synaptic weight, and the ratio α is the same as that in Equation (2). Δ*E*^*trans*^ and Δ*E*^*integ*^ are the metabolic energies consumed in synaptic transmission and dendritic integration over a period of time Δ*T*, respectively. It is clear that the left part of Equation (7) is equal to the product of the number and average weight of synaptic connections. It is well-known that synaptic connections are essential structures in neurons responsible for transmitting signals, and their number and strength determine the ability of neurons to transmit signals. Thus, the left part of Equation (7) actually characterizes the ability of neurons to transmit signals. The right part of Equation (7) undoubtedly reflects the energy metabolism in neurons. When signals flow to neurons, a large amount of energy is consumed to transmit and integrate these signals, and the metabolic energy consumed in these two processes depends on the ability of neurons to transmit signals. At a given time, a stronger ability of neurons to transmit signals corresponds to more metabolic energy consumed in synaptic transmission and dendritic integration, and vice versa. Obviously, the equation indicates that the ability of neurons to transmit signals should match their energy metabolism. Because this ability strongly depends on synaptic connections in neurons, we can further conclude from this equation that the synaptic connections in neurons should match their energy metabolism.

Our simulation results may provide some insight regarding the answer to the questions, why do neurons have thousands of synapses, and what factors affect the number of synaptic connections? (Hawkins and Ahmad, 2016) According to the above conclusions, the number of synaptic connections of neurons cannot increase infinitely. In the case that the metabolic energy balance remains constant, infinitely increasing the number of synaptic connections would inevitably result in the weights approaching zero. Synaptic connections whose weights are close to zero have weak information transmission capabilities. In contrast, if the number of synaptic connections is sacrificed for robust information transmission capabilities, this in turn leads to a decrease in the diversity of information received by neurons (Hawkins and Ahmad, 2016). Therefore, the number of synaptic connections owned by neurons depends on several factors such as the level of metabolic energy supply, the strength of synaptic connections, and the diversity of information.

### Network density is also an important factor for the energy balance in neuronal networks

After investigating the effects of metabolic energy on synaptic connections in individual neurons, we subsequently extend the simulation from individual neurons to neuronal networks with different numbers of neurons. The simulation results reveal that there is a roughly constant quantitative relationship among the number of neurons in the network, the average of synaptic weight in the entire network, and the average of the ratio of the energy consumed in synaptic transmission to the energy consumed in synaptic transmission and dendritic integration. In detail, while keeping the average ratio unchanged, the number of neurons in the network is inversely proportional to the average of synaptic weight of the entire network. This conclusion is an extension of the conclusion derived from the simulation of individual neurons.

To obtain more general synaptic organization rules, we analyse the simulation results from the perspective of graph theory. According to graph theory, neurons and synaptic connections can be regarded as nodes and edges in the network, respectively, and the number of synaptic connections of neurons is equivalent to the degree of nodes. It is worth noting that the neurons in the neuronal network defined by us in this paper are organized in a full connection manner. This means that as the number of neurons in the neuronal network increases, the degree of nodes also increases. However, in real biological neuronal networks, each neuron is coupled to only certain number of neurons, which is much smaller than the total number of neurons in the network (Golomb and Hansel, 2000). That is, the neuronal network is actually a non-fully connected network. For the convenience of discussion, the concept of network density is introduced here. The density of the network can be characterized by the average degree 〈*k*〉 of the network, which is the ratio of the number of edges to the number of nodes in the network. The greater the average degree of the neuronal network, the larger its density; otherwise, the smaller its density. Assuming a non-fully connected neuronal network, when we keep the average ratio unchanged and simultaneously increase the number of neurons and decrease the average degree of the neuronal network, the number and average weights of synaptic connections in the network do not change significantly. Therefore, the relations among average degree, the number of neurons, and the average of synaptic weights in the neuronal network can be mathematically described as follows:

(8) $$\langle k\rangle \cdot N\cdot \bar{w}\propto \frac{1}{N}\cdot \sum_{j=1}^{N}\frac{\Delta E_{j}^{trans}}{\Delta E_{j}^{trans}+\Delta E_{j}^{integ}}=\bar{\alpha }$$

where 〈*k*〉, *N*, and $\bar{w}$ represent the average degree, the number of neurons in the network, and the average weight of all synaptic connections in the neuronal network, respectively. $\bar{\alpha }$ represents the average of the ratio α of all neurons in the neuronal network. $\Delta E_{j}^{trans}$ and $\Delta E_{j}^{integ}$ represent the total energy consumed in synaptic transmission and dendritic integration in the *j-th* neuron over a period of time Δ*T*, respectively. Similar to individual neurons, the number and strength of synaptic connections in the neuronal network also determine the ability of the neuronal network to transmit signals. Thus, the left part of Equation (8) actually characterizes the ability of the neuronal network to transmit signals, and the right part reflects the energy metabolism in the network. When signals flow in the neuronal network, a large amount of metabolic energy is consumed to transmit and integrate these signals, and the metabolic energy consumed in these two processes depends on the ability of the neuronal network to transmit signals. At a given time, a stronger ability of the neuronal network to transmit signals corresponds to more metabolic energy consumed in synaptic transmission and dendritic integration, and vice versa. According to this equation, many conclusions similar to those obtained from the simulation results of individual neurons can also be obtained. For example, the ability of the neuronal network to transmit signals and the synaptic connections in the neuronal network should match the energy metabolism in the network. Furthermore, the more important inference we obtain is that in addition to the number and strength of synaptic connections discussed above, the density of the network is also associated with the energy metabolism in the network. Obviously, in the case of keeping the number of neurons in the neuronal network unchanged, when the average degree of the neuronal network increases, the strength of synaptic connections in the network should be reduced to maintain the energy balance. Otherwise, failure of the energy balance occurs.

Currently, increasing studies indicate that neurological disorders, such as Parkinson disease, Alzheimer's disease, and autism, and cognitive dysfunction are mainly caused by the pathological changes in neuronal networks (Belmonte et al., 2004; Crossley et al., 2014; Stam, 2014). Patients with these diseases exhibit failure of the energy metabolism of neuronal networks (Kapogiannis and Mattson, 2011; Pathak et al., 2013; Hillary and Grafman, 2017). The conclusions obtained in this paper may, to some extent, show how abnormalities in neuronal networks lead to failure of the energy metabolism of neuronal networks. According to our conclusions, the density of neuronal networks, the number of neurons in neuronal networks, and the strength of synaptic connections are associated with the energy metabolism of neuronal networks. When any one of these properties changes, the others can change accordingly to maintain the normal energy metabolism of the network. However, the adaptability of these properties is limited. When some of these properties change abnormally and exceed the adaptability of other properties, failure of the energy metabolism of neuronal networks occurs. As we know, the neuronal networks in the brain contain several tens of billions of neurons, and energy supply is limited. Even a slight change in the average degree of a local brain region can result in a large impact on energy metabolism in that brain region.

Note that the ratio of the number of excitatory neurons to the number inhibitory neurons is fixed at 4:1 in the network in this paper. In fact, excitatory neurons and inhibitory neurons account for 70–80 and 20–30% of all neocortical neurons, respectively (Markram et al., 2004). In the neural system, regardless of the level of excitation, the inhibitory system can automatically scale its output to provide matching opposition across a large dynamic range (Guo et al., 2016a). The inhibitory neurons in the network of this paper receive external signals and can balance the activity intensity of the entire network. Therefore, when the ratio of excitatory to inhibitory neurons changes, the activity intensity and energy consumption of the network will also change accordingly.

### Implicit influences of field coupling and body temperature on the energy metabolism

In this paper, synaptic connections are regarded as the only bridge for signal transmission between neurons in the neuronal network. In addition, some studies argue that field coupling could be another effective way for signal transmission because field coupling can realize phase synchronization between neurons (Ma et al., 2017). Further studies indicate that the synchronization degree is dependent on the coupling intensity and weight, and that the synchronization can be modulated by field coupling (Guo et al., 2017). During the movement, the charged ions in the magnetic field need consume energy to overcome the resistance from the cytoplasm, which may be one of the ways in which metabolic energy is consumed in field coupling. Therefore, it can be inferred that in addition to synaptic connections, part of the metabolic energy in the network may be also converted into magnetic field energy and even electric field energy during signal transmission.

The experimental results in this paper show that energy metabolism could constrain the topology of the neuronal network. Thus, it can be further inferred that the topology of the neuronal network is also related to the factors that can regulate energy metabolism in the brain. Some studies indicate that changes in body temperature can affect the metabolic rate and consequently the evolution of the brain (White et al., 2006). For example, warm temperatures can promote a variety of biochemical processes, and for a given body mass, warmer living conditions should result in larger brains. A larger brain means that the topology of the neuronal network in the brain is more complex, which is often associated with greater intelligence, better tool-making skills, and other enhanced characteristics. In addition, a warm body temperature can facilitate energy efficient cortical action potentials (Yu et al., 2012), and energy-efficient population coding constrains the size of a neuronal system (Yu et al., 2016). Therefore, it can be inferred that the topology of the neuronal network may be indirectly regulated by body temperature via the energy metabolism in the brain.

## Author contributions

YY and TF conceived the project and designed the experiments. All authors contributed to the development of the concepts presented in the paper. YY performed the experiments. YY performed the data analysis. All authors helped write the manuscript.

### Conflict of interest statement

The authors declare that the research was conducted in the absence of any commercial or financial relationships that could be construed as a potential conflict of interest.

## References

[B1] Alle H. Roth A. Geiger J. R. P. (2009). Energy-efficient action potentials in hippocampal mossy fibers. Science 15, 130–131. 10.1126/science.1174331 19745156

[B2] Attwell D. Laughlin S. B. (2001). An energy budget for signaling in the grey matter of the brain. J. Cereb. Blood Flow Metab. 21, 1133–1145. 10.1097/00004647-200110000-00001 11598490

[B3] Belmonte M. K. Allen G. Beckelmitchener A. Boulanger L. M. Carper R. A. Webb S. J. (2004). Autism and abnormal development of brain connectivity. J. Neurosci. 24, 9228–9231. 10.1523/JNEUROSCI.3340-04.2004 15496656 PMC6730085

[B4] Bialek W. Van Steveninck R. D. R. Tishby N. (2006). Efficient representation as a design principle for neural coding and computation, in International Symposium on Information Theory, 659–663. 10.1109/ISIT.2006.261867

[B5] Bohte S. M. Kok J. N. La Poutre H. (2002). Error-backpropagation in temporally encoded networks of spiking neurons. Neurocomputing 48, 17–37. 10.1016/S0925-2312(01)00658-0

[B6] Caporale N. Dan Y. (2008). Spike timing-dependent plasticity: a Hebbian learning rule. Annu. Rev. Neurosci. 31, 25–46. 10.1146/annurev.neuro.31.060407.125639 18275283

[B7] Corty M. M. Freeman M. R. (2013). Architects in neural circuit design: glia control neuron numbers and connectivity. J. Cell Biol. 203, 395–405. 10.1083/jcb.201306099 24217617 PMC3824021

[B8] Crossley N. A. Mechelli A. Scott J. Carletti F. Fox P. T. Mcguire P.. (2014). The hubs of the human connectome are generally implicated in the anatomy of brain disorders. Brain 137(Pt 8), 2382–2395. 10.1093/brain/awu132 25057133 PMC4107735

[B9] Davis G. W. (2006). Homeostatic control of neural activity: from phenomenology to molecular design. Annu. Rev. Neurosci. 29, 307–323. 10.1146/annurev.neuro.28.061604.135751 16776588

[B10] De Pittà M Brunel N. Volterra A. (2016). Astrocytes: orchestrating synaptic plasticity? Neuroscience 323, 43–61. 10.1016/j.neuroscience.2015.04.001 25862587

[B11] Foncelle A. Mendes A. Jedrzejewskaszmek J. Valtcheva S. Berry H. Blackwell K.. (2018). Modulation of spike-timing dependent plasticity: towards the inclusion of a third factor in computational models. Front. Comput. Neurosci. 12:49. 10.3389/fncom.2018.00049 30018546 PMC6037788

[B12] Frémaux N. Gerstner W. (2016). Neuromodulated spike-timing-dependent plasticity, and theory of three-factor learning rules. Front. Neural Circuits 9:85. 10.3389/fncir,.2015.00085 26834568 PMC4717313

[B13] Gerstner W. (1995). Time structure of the activity in neural network models. Phys. Rev. 51:738. 10.1103/PhysRevE.51.738 9962697

[B14] Golomb D. Hansel D. (2000). The number of synaptic inputs and the synchrony of large, sparse neuronal networks. Neural Comput. 12, 1095–1139. 10.1162/089976600300015529 10905810

[B15] Gu S. Pasqualetti F. Cieslak M. Telesford Q. K. Yu A. B. Kahn A. E.. (2015). Controllability of structural brain networks. Nat. Commun. 6:8414. 10.1038/ncomms9414 26423222 PMC4600713

[B16] Guo D. Chen M. Perc M. Wu S. Xia C. Zhang Y. (2016a). Firing regulation of fast-spiking interneurons by autaptic inhibition. Epl 114, 30001 10.1209/0295-5075/114/30001

[B17] Guo D. Wang Q. Perc M. (2012). Complex synchronous behavior in interneuronal networks with delayed inhibitory and fast electrical synapses. Phys. Rev. Statist. Nonlin. Soft Matter Phys. 85, 878–896. 10.1103/PhysRevE.85.061905 23005125

[B18] Guo D. Wu S. Chen M. Matja,Ž P. Zhang Y. Ma J.. (2016b). Regulation of irregular neuronal firing by autaptic transmission. Sci. Rep. 6:26096. 10.1038/srep26096 27185280 PMC4869121

[B19] Guo S. Xu Y. Wang C. Jin W. Hobiny A. Ma J. (2017). Collective response, synapse coupling and field coupling in neuronal network. Chaos Solitons Fractals 105, 120–127. 10.1016/j.chaos.2017.10.019

[B20] Hasenstaub A. Otte S. Callaway E. Sejnowski T. J. (2010). Metabolic cost as a unifying principle governing neuronal biophysics. Proc. Natl. Acad. Sci. USA. 107, 12329–12334. 10.1073/pnas.0914886107 20616090 PMC2901447

[B21] Hattori R. Kuchibhotla K. V. Froemke R. C. Komiyama T. (2017). Functions and dysfunctions of neocortical inhibitory neuron subtypes. Nat. Neurosci. 20:1199. 10.1038/nn.4619 28849791 PMC7082034

[B22] Hawkins J. Ahmad S. (2016). Why neurons have thousands of synapses, a theory of sequence memory in neocortex. Front. Neural Circuits 10:23. 10.3389/fncir.2016.00023 27065813 PMC4811948

[B23] Hebb D. O. (1949). The Organization of Behavior. New York, NY: Wiley.

[B24] Hillary F. G. Grafman J. H. (2017). Injured Brains and Adaptive networks: the benefits and costs of hyperconnectivity. Trends Cogn. Sci. 21:385. 10.1016/j.tics.2017.03.003 28372878 PMC6664441

[B25] Howarth C. Gleeson P. Attwell D. (2012). Updated energy budgets for neural computation in the neocortex and cerebellum. J. Cereb. Blood Flow Metab. 32:1222. 10.1038/jcbfm.2012.35 22434069 PMC3390818

[B26] Howarth C. Peppiatt W. C. D. (2010). The energy use associated with neural computation in the cerebellum. J. Cereb. Blood Flow Metab. 30, 403–414. 10.1038/jcbfm.2009.231 19888288 PMC2859342

[B27] Izhikevich E. M. (2004). Which model to use for cortical spiking neurons? Neural Netw. IEEE Trans. 15, 1063–1070. 10.1109/TNN.2004.832719 15484883

[B28] Kapogiannis D. Mattson M. P. (2011). Disrupted energy metabolism and neuronal circuit dysfunction in cognitive impairment and Alzheimer's disease. Lancet Neurol. 10, 187–198. 10.1016/S1474-4422(10)70277-5 21147038 PMC3026092

[B29] Laughlin S. B. (2001). Energy as a constraint on the coding and processing of sensory information. Curr. Opin. Neurobiol. 11, 475–480. 10.1016/S0959-4388(00)00237-3 11502395

[B30] Ma J. Mi L. Zhou P. Xu Y. Hayat T. (2017). Phase synchronization between two neurons induced by coupling of electromagnetic field. Appl. Math. Comput. 307, 321–328. 10.1016/j.amc.2017.03.002

[B31] Magee J. C. (2000). Dendritic integration of excitatory synaptic input. Nat. Rev. Neurosci. 1, 181–190. 10.1038/35044552 11257906

[B32] Magistretti P. Allaman I. (2015). A cellular perspective on brain energy metabolism and functional imaging. Neuron 86, 883–901. 10.1016/j.neuron.2015.03.035 25996133

[B33] Markram H. Toledo-Rodriguez M. Wang Y. Gupta A. Silberberg G. Wu C. (2004). Interneurons of the neocortical inhibitory system. Nat. Rev. Neurosci. 5:793. 10.1038/nrn1519 15378039

[B34] McDonnell M. D. Abbott D. (2009). What is stochastic resonance? Definitions, misconceptions, debates, and its relevance to biology. PLoS Comput. Biol. 5:e1000348. 10.1371/journal.pcbi.1000348 19562010 PMC2660436

[B35] Medaglia J. D. Lynall M. E. Bassett D. S. (2015). Cognitive network neuroscience. J. Cogn. Neurosci. 27, 1471–1491. 10.1162/jocn 25803596 PMC4854276

[B36] Mitsushima D. Sano A. Takahashi T. (2013). A cholinergic trigger drives learning-induced plasticity at hippocampal synapses. Nat. Commun. 4:2760. 10.1038/ncomms3760 24217681 PMC3831287

[B37] Nessler B. Pfeiffer M. Buesing L. Maass W. (2013). Bayesian computation emerges in generic cortical microcircuits through spike-timing-dependent plasticity. PLoS Comput. Biol. 9:e1003037. 10.1371/journal.pcbi.1003037 23633941 PMC3636028

[B38] Neves G. Cooke S. F. Bliss T. V. (2008). Synaptic plasticity, memory and the hippocampus: a neural network approach to causality. Nat. Rev. Neurosci. 9, 65–75. 10.1038/nrn2303 18094707

[B39] Niven J. E. Laughlin S. B. (2008). Energy limitation as a selective pressure on the evolution of sensory systems. J. Exp. Biol. 211, 1792–1804. 10.1242/jeb.017574 18490395

[B40] Pathak D. Berthet A. Nakamura K. (2013). Energy failure: does it contribute to neurodegeneration? Ann. Neurol. 74, 506–516. 10.1002/ana.24014 24038413 PMC4092015

[B41] Picciotto M. R. Higley M. J. Mineur Y. S. (2012). Acetylcholine as a neuromodulator: cholinergic signaling shapes nervous system function and behavior. Neuron 76, 116–129. 10.1016/j.neuron.2012.08.036 23040810 PMC3466476

[B42] Raichle M. E. Mintun M. A. (2006). Brain work and brain imaging. Annu. Rev. Neurosci. 29, 449–476. 10.1146/annurev.neuro.29.051605.112819 16776593

[B43] Smith S. M. Fox P. T. Miller K. L. Glahn D. C. Fox P. M. Mackay C. E.. (2009). Correspondence of the brain's functional architecture during activation and rest. Proc. Natl. Acad. Sci. U.S.A. 106, 13040–13045. 10.1073/pnas.0905267106 19620724 PMC2722273

[B44] Spruston N. (2008). Pyramidal neurons: dendritic structure and synaptic integration. Nat. Rev. Neurosci. 9, 206–221. 10.1038/nrn2286 18270515

[B45] Stam C. J. (2014). Modern network science of neurological disorders. Nat. Rev. Neurosci. 15:683. 10.1038/nrn3801 25186238

[B46] Stuart G. J. Spruston N. (2015). Dendritic integration: 60 years of progress. Nat. Neurosci. 18:1713. 10.1038/nn.4157 26605882

[B47] Südhof T. C. (2013). Neurotransmitter Release: The last millisecond in the life of a synaptic vesicle. Neuron 80, 675–690. 10.1016/j.neuron.2013.10.022 24183019 PMC3866025

[B48] Sussillo D. (2014). Neural circuits as computational dynamical systems. Curr. Opin. Neurobiol. 25, 156–163. 10.1016/j.conb.2014.01.008 24509098

[B49] Sussillo D. Abbott L. F. (2012). Transferring learning from external to internal weights in Echo-State networks with sparse connectivity. PLoS ONE 7:e37372. 10.1371/journal.pone.0037372 22655041 PMC3360031

[B50] Sweatt J. D. (2016). Neural plasticity and behavior – sixty years of conceptual advances. J. Neurochem. 139, 179–199. 10.1111/jnc.13580 26875778

[B51] Turrigiano G. (2007). Homeostatic signaling: the positive side of negative feedback. Curr. Opin. Neurobiol. 17, 318–324. 10.1016/j.conb.2007.04.004 17451937

[B52] Wang Q. Chen G. Perc M. (2011). Synchronous bursts on scale-free neuronal networks with attractive and repulsive coupling. PLoS ONE 6:e15851. 10.1371/journal.pone.0015851 21253015 PMC3017050

[B53] Waterson M. J. Horvath T. L. (2015). Neuronal regulation of energy homeostasis: beyond the hypothalamus and feeding. Cell Metab. 22, 962–970. 10.1016/j.cmet.2015.09.026 26603190

[B54] White C. R. Phillips N. F. Seymour R. S. (2006). The scaling and temperature dependence of vertebrate metabolism. Biol. Lett. 2, 125–127. 10.1098/rsbl.2005.0378 17148344 PMC1617203

[B55] Wu L. G. Hamid E. Shin W. Chiang H. C. (2014). Exocytosis and endocytosis: modes, functions, and coupling mechanisms^*^. Annu. Rev. Physiol. 76:301 10.1146/annurev-physiol-021113-170305 24274740 PMC4880020

[B56] Xu Y. Jia Y. Ma J. Hayat T. Alsaedi A. (2018). Collective responses in electrical activities of neurons under field coupling. Sci. Rep. 8:1349. 10.1038/s41598-018-19858-1 29358677 PMC5778049

[B57] Yu L. Yu Y. (2017). Energy-efficient neural information processing in individual neurons and neuronal networks. J. Neurosci. Res. 95, 2253–2266. 10.1002/jnr.24131 28833444

[B58] Yu L. Zhang C. Liu L. Yu Y. (2016). Energy-efficient population coding constrains network size of a neuronal array system. Sci. Rep. 6:1939. 10.1038/srep19369 26781354 PMC4725972

[B59] Yu Y. Herman P. Rothman D. L. Agarwal D. Hyder F. (2017). Evaluating the gray and white matter energy budgets of human brain function. J. Cereb. Blood Flow Metab. 271678X17708691. 28589753 10.1177/0271678X17708691 PMC6092772

[B60] Yu Y. Hill A. P. Mccormick D. A. (2012). Warm body temperature facilitates energy efficient cortical action potentials. PLoS Comput. Biol. 8:e1002456. 10.1371/journal.pcbi.1002456 22511855 PMC3325181

[B61] Yu Y. Karbowski J. Sachdev R. N. Feng J. (2014). Effect of temperature and glia in brain size enlargement and origin of allometric body-brain size scaling in vertebrates. BMC Evol. Biol. 14:178. 10.1186/s12862-014-0178-z 25277168 PMC4193995

[B62] Yuan Y. Huo H. Fang T. (2018). Effects of metabolic energy on synaptic transmission and dendritic integration in pyramidal neurons. Front. Comput. Neurosci. 12:79. 10.3389/fncom.2018.00079 30319383 PMC6168642

