# Supplementary material for: Constraints of Metabolic Energy on the Number of Synaptic Connections of Neurons and the Density of Neuronal Networks
Source: Front Comput Neurosci. 2018 Nov 20;12:91. doi: 10.3389/fncom.2018.00091 (PMC6256250; doi:10.3389/fncom.2018.00091)
Supplement: Supplementary file 1 [file Presentation_1.pdf]

Constraints of metabolic energy on the number of synaptic connections of neurons and the density of neuronal networks

Ye Yuan[1,2], Hong Huo[1,2*], Peng Zhao[1,2], Jian Liu[1,2], Jiaxing Liu[1,2], Fu Xing[1,2] & Tao Fang[1,2*]

[1]Department of Automation, Shanghai Jiao Tong University, Shanghai 200240, China

[2]Key Laboratory of System Control and Information Processing, Ministry of Education, Shanghai 200240, China

Supplementary materials

Leaky integrate-and-fire neuron. The leaky integrate-and-fire neuron is one of the most widely used models in computational neuroscience(Izhikevich, 2004). It can be stimulated either by an external current or by synaptic inputs from presynaptic neurons. The leaky integrate-and-fire neuron can be described as follows:

$$u(t) = -\tau_m \cdot \dot{u}(t) + R \cdot I(t),\ if\ u(t) > V_{th},\ u(t) \leftarrow 0 \tag{1}$$

where $u(t)$ and $I(t)$ represent the membrane potential and driving current of neurons, respectively. R represents the resistance coefficient, and τ_m denotes the change rate of the membrane potential. V_{th} is the threshold of the membrane potential. In the case of the same driving current, increasing the resistance coefficient or the change rate can both accelerate changes in the membrane potential. Generally, the change rate is considered a constant. The driving current can be described as follows:

$$I(t) = \rho \cdot \sum_{i=1}^{O} \sum_{j=1}^{P} w_i \cdot \frac{t - t_j}{\tau_{sys}} \cdot e^{-\frac{t-t_j}{\tau_{sys}}}, (t \geq t_j) \tag{2}$$

where O represents the number of synaptic connections, and P the number of spikes transmitted to the neuron through the *i-th* synaptic connection before time t. w_i represents the weight of the *i-th* synaptic connection, and t_j represents the time of the *j-th* spike signal arriving at the neuron. τ_{sys} is time constant, and ρ is gain coefficient of synaptic signals. The default values of the parameters in the leaky integrate-and-fire neuron model are: $R = 1.0\times10^6\ \Omega$, $\tau_m = 100$ ms, $V_{th} = 85.0$ mV, $\tau_{sys} = 20$ ms, and $\rho = 8.0\times10^{-7}$, respectively.

Spike-timing-dependent plasticity rules. Many experimental and theoretical studies support the

Hebbian postulate and suggest that changes in synaptic weights depend on the activities of pre- and postsynaptic neurons(Sjöström et al., 2008). The spike-timing-dependent plasticity (STDP) rules are abstracted from the Hebbian postulate and have been considered the most biologically plausible synaptic plasticity rules(Song et al., 2000). The STDP learning rules can be described as follows (Morrison et al., 2008):

$$H(pre, post) = \begin{cases} \eta(1-w)^{\mu} \cdot e^{-\frac{|\Delta t|}{\tau_+}}, & if\, \Delta t > 0 \\ -\eta\sigma w^{\mu} \cdot e^{-\frac{|\Delta t|}{\tau_-}}, & if\, \Delta t \leq 0 \end{cases} \tag{3}$$

where η is the learning rate, and σ is an asymmetry parameter. τ_+ and τ_- represent the time constants of long-term potentiation and depression, respectively. As defined in Equation (6), $\Delta t = t_{post} - t_{pre}$ is the temporal difference between the post- and presynaptic spikes. The parameter $\mu (0 \leq \mu \leq 1)$ reflects the extent to which synaptic plasticity is affected by the current synaptic strength. A choice of $\mu = 0$ leads to additive STDP, and a choice of $\mu = 1$ leads to multiplicative STDP. Here, we chose $\mu = 1$ to ensure that the network has better robustness. The default values of the parameters in Equation (11) in this paper are: $\eta = 0.001$, $\mu = 1$, $\sigma = 1$, and $\tau_+ = \tau_- = 10.0$ ms, respectively.

References

Izhikevich, E.M. (2004). Which model to use for cortical spiking neurons? *Neural Networks IEEE Transactions on* 15(5), 1063-1070.

Morrison, Abigail, Diesmann, Markus, Gerstner, and Wulfram (2008). Phenomenological models of synaptic plasticity based on spike timing. *Biological Cybernetics* 98(6), 459-478.

Sjöström, P.J., Rancz, E.A., Roth, A., and Häusser, M. (2008). Dendritic excitability and synaptic plasticity. *Physiological Reviews* 88(2), 769-840.

Song, S., Miller, K.D., and Abbott, L.F. (2000). Competitive Hebbian learning through spike-timing-dependent synaptic plasticity. *Nature Neuroscience* 3(9), 919-926.
